# Fast and Stable Zinc Anode-Based Electrochromic Displays Enabled by Bimetallically Doped Vanadate and Aqueous Zn^2+^/Na^+^ Hybrid Electrolytes

**DOI:** 10.1007/s40820-023-01209-z

**Published:** 2023-10-17

**Authors:** Zhaoyang Song, Bin Wang, Wu Zhang, Qianqian Zhu, Abdulhakem Y. Elezzabi, Linhua Liu, William W. Yu, Haizeng Li

**Affiliations:** 1 College of Chemistry and Chemical Engineering, Qingdao University, Qingdao, 266071 People’s Republic of China; 2 Optics and Thermal Radiation Research Center, Institute of Frontier and Interdisciplinary Science, Shandong University, Qingdao, 266237 People’s Republic of China; 3 School of Chemistry and Chemical Engineering, Shandong University, Jinan, 250100 People’s Republic of China; 4 Ultrafast Optics and Nanophotonics Laboratory, Department of Electrical and Computer Engineering, University of Alberta, Edmonton, AB T6G 2V4 Canada

**Keywords:** Vanadates, Hybrid electrolytes, Displays, Electrochromic

## Abstract

Vanadates are a class of the most promising electrochromic materials for displays as their multicolor characteristics. However, the slow switching times and vanadate dissolution issues of recently reported vanadates significantly hinder their diverse practical applications. Herein, novel strategies are developed to design electrochemically stable vanadates having rapid switching times. We show that the interlayer spacing is greatly broadened by introducing sodium and lanthanum ions into V_3_O_8_ interlayers, which facilitates the transportation of cations and enhances the electrochemical kinetics. In addition, a hybrid Zn^2+^/Na^+^ electrolyte is designed to inhibit vanadate dissolution while significantly accelerating electrochemical kinetics. As a result, our electrochromic displays yield the most rapid switching times in comparison with any reported Zn-vanadate electrochromic displays. It is envisioned that stable vanadate-based electrochromic displays having video speed switching are appearing on the near horizon.

**Supplementary Information:**

The online version contains supplementary material available at 10.1007/s40820-023-01209-z.

## Highlights

- La^3+^/Na^+^ bimetallically doped vanadate, designed for the first time, is promising in many electrochemical applications (e.g., batteries, electrochromics).
- This is the first report of electrochromic displays employing bimetallically doped vanadate.
- It is demonstrated for the first time that zinc dendrites and vanadate dissolution are significantly inhibited by employing an aqueous hybrid Zn^2+^/Na^+^ electrolyte.

**Supplementary Information:**

The online version contains supplementary material available at 10.1007/s40820-023-01209-z.

## Introduction

With the continuous advancement of modern displays, electrochromic (EC) technology is attracting emerging attention for its low energy consumption, easy integration, and enhanced visualization. This technology exhibits compelling potential for applications in nonemissive displays [1], smart windows [2], camouflages [3–6], and wearable electronic devices [7–11]. While organic electrochromic materials are regarded as a representative paradigm for electrochromic displays due to their rapid switching times and vivid colors [12, 13], their inferior thermal and chemical stabilities greatly hinder their real-world applications and potential commercialization. Consequently, the exploration of stable inorganic electrochromic materials having rapid switching times is regarded as a paradigm shift within the electrochromic community.

Vanadate oxide (V_2_O_5_), a classic inorganic electrochromic material, has recently intrigued significant attention due to its bipolar properties, particularly in display applications [14]. However, the strong electrostatic interaction between cations and V_2_O_5_ lattice during redox reactions results in slow switching times and poor cycling stability due to inferior structural stability [15, 16]. In order to tackle these issues and boost the structural stability of V_2_O_5_, sodium ion-stabilized vanadate oxide (SVO) nanorods were designed for Zn^2+^-triggered electrochromic displays [17]. Nevertheless, the SVO electrode showed limited structural stability and switching speed, as the enlargement of interlayer spacing is highly constrained by the restricted concentration of Na^+^. Therefore, the introduction of additional intercalated cations in layered SVO (i.e., bimetallically doped vanadate) is supposed to be an efficient strategy to solve the aforementioned shortcomings. Considering that La^3+^ shares a similar radius with Na^+^, the intercalation of La^3+^ into SVO layers offers a viable approach to further expand the interlayer spacing of V_3_O_8_. Similar to Na^+^, multivalent La^3+^ could serve as interlayer pillars in the SVO lattice to stabilize the structure and enhance ion-conducting properties. Furthermore, the multivalent La^3+^, with their multiple charges, could significantly weaken the electrostatic interaction between guest cations and layer structures, thereby boosting the structural stability of the vanadates [18].

Recently, aqueous zinc anode-based electrochromic devices, established by our group, have attracted much attention from many researchers due to their great potential in the field of smart electronics [19–23]. Nowadays, zinc anode-based electrochromic devices for display applications were also developed rapidly as their vivid color hues and compelling 2D CIE color space tunability [17, 24, 25]. However, vanadate dissolution is another key challenge for constructing stable aqueous zinc anode-based electrochromic displays. Most recently reported zinc anode-based electrochromic displays employed aqueous ZnSO_4_ electrolytes [17, 26, 27], which brings irreversible zinc anode reactions and dissolution of the vanadate, due to the side effects of SO_4_^2−^. Hence, the study of decent aqueous electrolyte systems, including the exploration of applicable cations and anions, warrants further investigation.

Herein, La^3+^/Na^+^ bimetallically doped vanadate (LaSVO) is designed and employed for electrochromic displays for the first time. The further intercalation of La^3+^ into SVO lattice significantly broadened the interlayer spacing without changing the phase structure of SVO, which enables a superior ionic diffusion coefficient. Remarkably, the employment of an aqueous hybrid electrolyte comprising ZnCl_2_ and NaCl endows the high reversibility of zinc anode and effectively inhibits the dissolution of vanadate. Such findings empower the Zn//ZnCl_2_-NaCl//LaSVO electrochromic display platform exhibits the most compelling switching times (4.5/8.8 s for coloration and bleaching, respectively) and exceptional stability. As a proof of concept, a prototype zinc anode-based electrochromic display is constructed. Such a display possesses a 1.44 V open-circuit potential (OCP), which is capable of spontaneously switching colors from orange to green via lighting a 0.2 V LED.

## Experimental Section

### Materials

All chemicals were of analytical grade and were used without further purification. Sodium chloride (NaCl, 99.5%), zinc foil (Zn, 99.9%), vanadate oxide (V_2_O_5_, 99%), and polyvinylpyrrolidone (PVP, Mw ~ 1,300,000) were purchased from Macklin Biochemical Technology Co. Ltd. Zinc chloride (ZnCl_2_, 98.0%) was purchased from Hengxing Chemical Reagent Co. Ltd. Lanthanum nitrate hexahydrate (La(NO_3_)_3_·6H_2_O, 99%) and hydrochloric acid (HCl, 36.0–38.0%) were purchased from Sinopharm Chemical Reagent Co. Ltd. Hydroxyethyl cellulose (HEC, Mw ~ 30,000) was purchased from Usolf Chemical Co. Ltd. ITO glass were purchased from Zhuhai Kaivo Glass Co. Ltd. Scotch magic tape (12.7 mm × 10 m) was purchased from 3 M Material Technology (Suzhou) Co.

### Synthesis of La-doped Sodium Vanadate Oxide (LaSVO) Nanorods

First, 150 mL of sodium chloride aqueous solution (2 M) was prepared at room temperature, and then, 10 g of commercial V_2_O_5_ powder was added into the solution and being stirred for more than 96 h until form a jacinth suspension. Next, 10 g of lanthanum nitrate hexahydrate was added into the suspension, and being stirred for 24 h. Then, the suspension was purified by adding deionized water and subjected to centrifugation with deionized water for six times. Finally, the product was diluted with deionized water to prepare a colloid with a concentration of 10 mg mL^−1^.

### Fabrication of LaSVO Electrodes

The LaSVO/HEC paste with a specific viscosity must be prepared before bar-coating. LaSVO/HEC paste was obtained by adding 0.7 g HEC to 30 mL LaSVO colloid (10 mg mL^−1^) at room temperature and being stirred for over 6 h. After that, the LaSVO/HEC paste was bar-coated onto the clean ITO glass with Scotch magic tape as a spacer for determining the thickness of the LaSVO film. Then, the bar-coated LaSVO/HEC electrodes were sequentially annealed at 100 °C for 2 h and 180 °C for 20 h. The thickness of the as-annealed LaSVO film was about 965 nm (Fig. S1).

### Assembly of LaSVO-Zn-LaSVO Electrochromic Displays

The PVP-based gel electrolyte was prepared by gradually adding 10 g of PVP powder into 40 mL of the hybrid aqueous solution comprising 0.1 M ZnCl_2_ and 1.8 M NaCl (pH controlled at approximately 5.3). The Zn-LaSVO electrochromic display was constructed by sandwiching a zinc frame between two LaSVO electrodes. The aforementioned PVP-based gel electrolyte was used as the electrolyte.

### Characterization

The crystal structures and morphology of the samples were examined by X-ray diffraction (XRD, Rigaku D/Max 2500/PC diffractometer with a graphite monochromator and Cu Kα radiation (*λ* = 0.15418 nm)), X-ray photoelectron spectroscopy (XPS) (PHI 5000 VersaProbe III), field emission scanning electron microscope (FESEM, FEI Quanta 250 FEG) and high-resolution transmission electron microscope (HRTEM, JEM-F200(HRP)).

### Optical and Electrochemical Measurements

All optical measurements were performed using a UV–Visible–NIR Spectrophotometer (UH5700). All electrochemical measurements were carried out using an electrochemical workstation (CHI-760E, CH Instruments, Shanghai, China) in a two-electrode configuration, using the electrochromic electrode as the working electrode, a Zn foil as the counter electrode and reference electrode. In situ optical transmittance as a function of the applied potential was obtained in a quartz cuvette recorded by the UV–Visible–NIR Spectrophotometer. ITO glass immersed in electrolyte was used as the baseline for measuring the transmittance of the electrodes. In this work, two types of solutions were used as electrolyte: ZnCl_2_ solution (1 M) and a hybrid aqueous solution comprised of 0.1 M ZnCl_2_ and 1.8 M NaCl. The pH value of the electrolyte was adjusted to be ~ 5.3 by dropping 12 M HCl.

## Results and Discussion

### Characterization of LaSVO Nanofibers

**Fig. 1:**
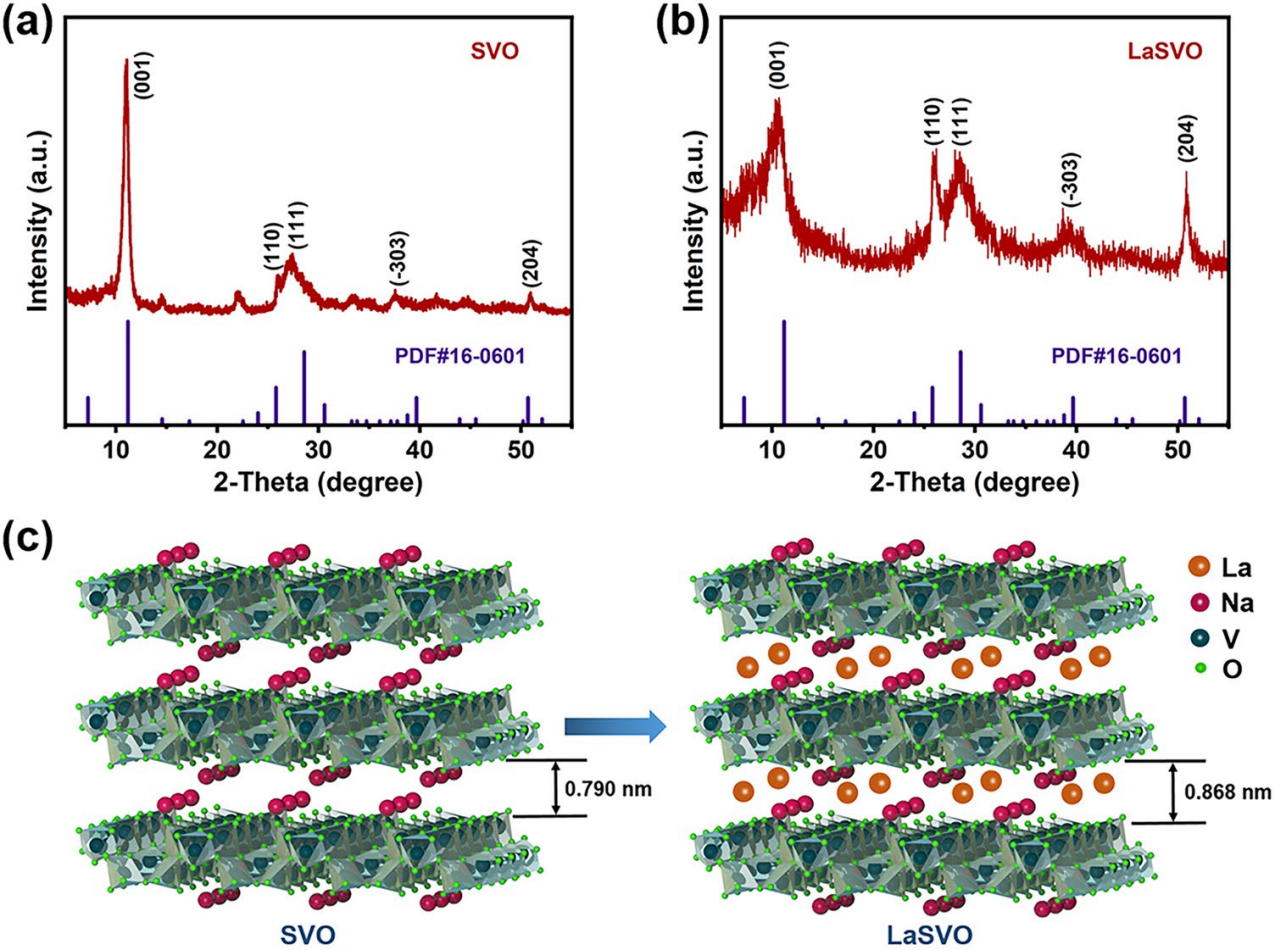
The comparison of SVO and LaSVO crystal structures. **a**, **b** XRD patterns of the as-prepared SVO and LaSVO. **c** Schematically illustration of the SVO and LaSVO crystal structures, Na^+^ or La^3+^ exists in the form of hydrated ions

The LaSVO nanorods were prepared by a modified liquid–solid stirring method at room temperature (See details in Sect. 2) [17]. The phase composition of LaSVO and SVO was analyzed via powder XRD. As depicted in the XRD patterns (Fig. 1a, b), the diffraction peaks of LaSVO and SVO are all accurately matched with the monoclinic NaV_3_O_8_·1.5H_2_O phase (JCPDS No. 16-0601). This means the intercalation of La^3+^ into the SVO lattice has no effect on the phase structure of the SVO, due to the almost identical radius between La^3+^ and Na^+^. Such a monoclinic SVO phase, having a layered structure within the (001) crystal plane, is favorable for cations transportation and electrochemical kinetics. Remarkably, the LaSVO shows a broadened lattice spacing of (001) crystal plane (i.e., interlayer spacing), according to Bragg’s law (2dsin*θ* = *nλ*). As calculated from XRD patterns, the intercalation of La^3+^ into SVO lattice expanded the interlay spacing from 0.79 to 0.868 nm (Fig. 1c). This enlarged lattice spacing will result in rapid response times and improved structural stability [28].

**Fig. 2:**
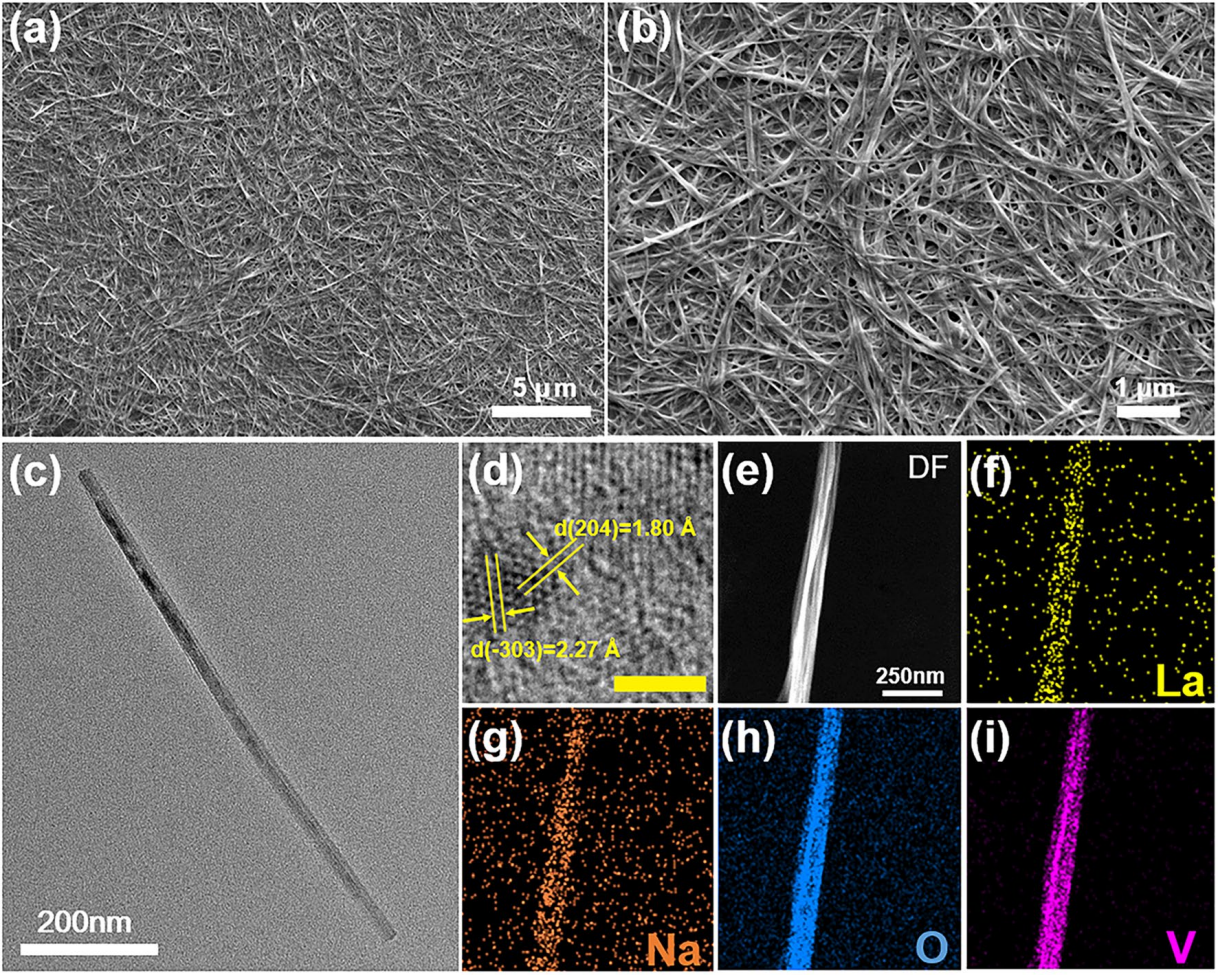
Morphological characterization of LaSVO. **a**, **b** Low magnification and high magnification SEM images of LaSVO nanofibers. **c** Bright-field (BF) TEM image of LaSVO nanofibers. **d** High-resolution TEM image of a LaSVO nanofiber depicting the lattice planes (scale bar: 2 nm). **e**–**i** Dark-field (DF) TEM image of LaSVO nanofibers and the corresponding elemental mapping images of La, Na, O, and V

To demonstrate the morphology of LaSVO and SVO, scanning electron microscopy (SEM) was employed. The LaSVO and SVO samples all exhibit a 1D fiber structure, as demonstrated in Fig. 2a, b and S2, which endows them being cross-linked into a fiber network when fabricating electrochromic electrodes [29]. The fiber networks are favorable for cations transportation between the electrolyte and electrochromic materials. Transmission electron microscopy (TEM) was also conducted to observe the crystallization and the aspect ratio of individual LaSVO nanofiber. The length of LaSVO nanofiber, as depicted in Fig. 2c, was determined to be approximately 800 nm with a diameter of roughly 25 nm, thereby exhibiting a high aspect ratio approximate of 30. High-resolution TEM image shows crystalline lattice spacings of 2.27 and 1.80 Å corresponding to the (− 303) and (204) crystal planes, respectively (Fig. 2d). Energy-dispersive X-ray spectroscopy (EDX) mapping affirms the presence and uniform distribution of La, Na, O, and V elements within LaSVO nanofibers (Fig. 2e–i). These results confirm that La^3+^ and Na^+^ have been intercalated into the V_3_O_8_ interlayers without changing the morphology.

### Inhibition of Zn Anode Dendrites and LaSVO Dissolution by Using an Aqueous Hybrid Zn^2+^/Na^+^ Electrolyte

**Fig. 3:**
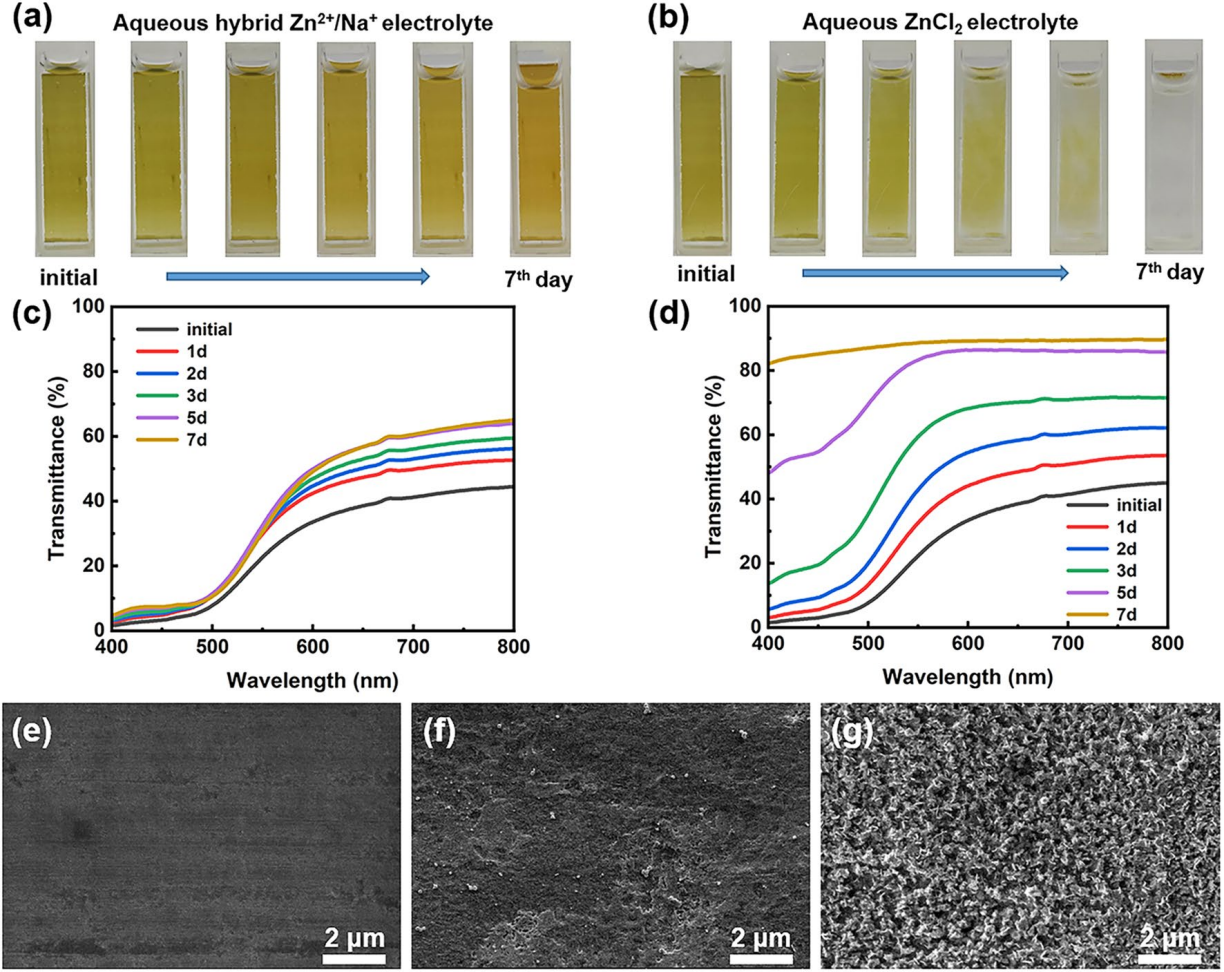
Hybrid electrolyte improving the stability of LaSVO electrode and Zn anode. Digital photos of LaSVO electrodes immersed in** a** hybrid ZnCl_2_–NaCl electrolyte and** b** pure ZnCl_2_ electrolyte, respectively. Transmission evolution of LaSVO film after being immersed in **c** the hybrid ZnCl_2_–NaCl electrolyte and **d** in the ZnCl_2_ electrolyte for seven days. SEM images of zinc anode in **e** initial state, after 1000 CV cycles **f** in the hybrid ZnCl_2_–NaCl electrolyte and **g** pure ZnCl_2_ electrolyte

With the excellent bimetallically doped vanadate (i.e., LaSVO) having broadened interlayer spacing being successfully prepared, we next investigated the appropriate electrolyte system for stable Zn-LaSVO electrochromic displays. While previous reports using aqueous ZnSO_4_ solution as electrolytes suffer zinc dendrites and vanadate dissolution [17, 27], a superior electrolyte system is of significant importance for constructing stable Zn-vanadate electrochromic displays. Considering that the vanadate dissolution is originated from unbalanced element concentration between electrochromic materials and electrolyte, the addition of Na^+^ into the electrolyte is supposed to change the dissolution equilibrium of Na^+^ from sodium vanadate electrodes and thus inhibiting the continuous sodium vanadate dissolution [30]. In addition, Na^+^ has a lower reduction potential than Zn^2+^, which could form a positively electrostatic shield around the Zn protuberances, thus avoiding the formation of Zn dendrites [31]. Furthermore, the presence of SO_4_^2−^ in electrolytes tends to form hydroxyl sulfate by-products on both sides of the cathode and anode [32], thus hindering long-term real-world applications. In this regard, we explored the dissolution and Zn dendrites inhibition performance by using an aqueous hybrid electrolyte comprised of 0.1 M ZnCl_2_ and 1.8 M NaCl. The selection of Cl^−^ anion in the current electrolyte system is because Cl^−^ facilitates the desolvation effect in aqueous ZnCl_2_ solutions [33]. As shown in Fig. 3a, the LaSVO electrode is slowly oxidized due to the presence of dissolved oxygen in the aqueous hybrid Zn^2+^/Na^+^ electrolyte [34], which oxidizes the LaSVO electrode and thus switches its color from pale yellow to orange. In contrast, the LaSVO electrode, immersed in the pure aqueous ZnCl_2_ electrolyte, was gradually dissolved into the electrolyte. The optical transmittance spectra of the LaSVO electrode in Fig. 3c, d affirm the above dissolution phenomenon. These results confirm that the addition of Na^+^ into the electrolyte alters the dissolution equilibrium of Na^+^ from LaSVO, thus effectively halting the ongoing dissolution process of LaSVO.

Additionally, the use of an aqueous hybrid electrolyte greatly diminishes the formation of Zn dendrites on the Zn anode. As shown in Fig. 3e, the surface of the zinc foil before cyclic voltammetry (CV) cycling was extremely smooth and flat. After 1000 CV cycles, the zinc foil in the hybrid electrolyte keeps smooth and flat without dendrites (Fig. 3f). In contrast, obvious dendrites are observed on zinc foil when cycled in pure aqueous ZnCl_2_ electrolyte (Fig. 3g). These results affirm that the aqueous hybrid electrolyte comprised of 0.1 M ZnCl_2_ and 1.8 M NaCl is promising for constructing stable Zn-vanadate aqueous electrochromic displays.

### Electrochemical and Electrochromic Performance of LaSVO Electrodes

With the careful selection of the LaSVO electrode having wide interlayer spacing, and the hybrid electrolyte system inhibiting vanadate dissolution as well as Zn dendrites formation, we then investigated the electrochromic performance of the LaSVO electrodes in the aforementioned hybrid electrolyte. Electrochemical and electrochromic measurements were performed using a two-electrode configuration where zinc foil was used as the anode and vanadate electrode as the cathode. The aqueous hybrid Zn^2+^/Na^+^ electrolyte (i.e., 0.1 M ZnCl_2_–1.8 M NaCl) was used in this platform and the pure ZnCl_2_ electrolyte (1 M) was used as the control sample.

**Fig. 4:**
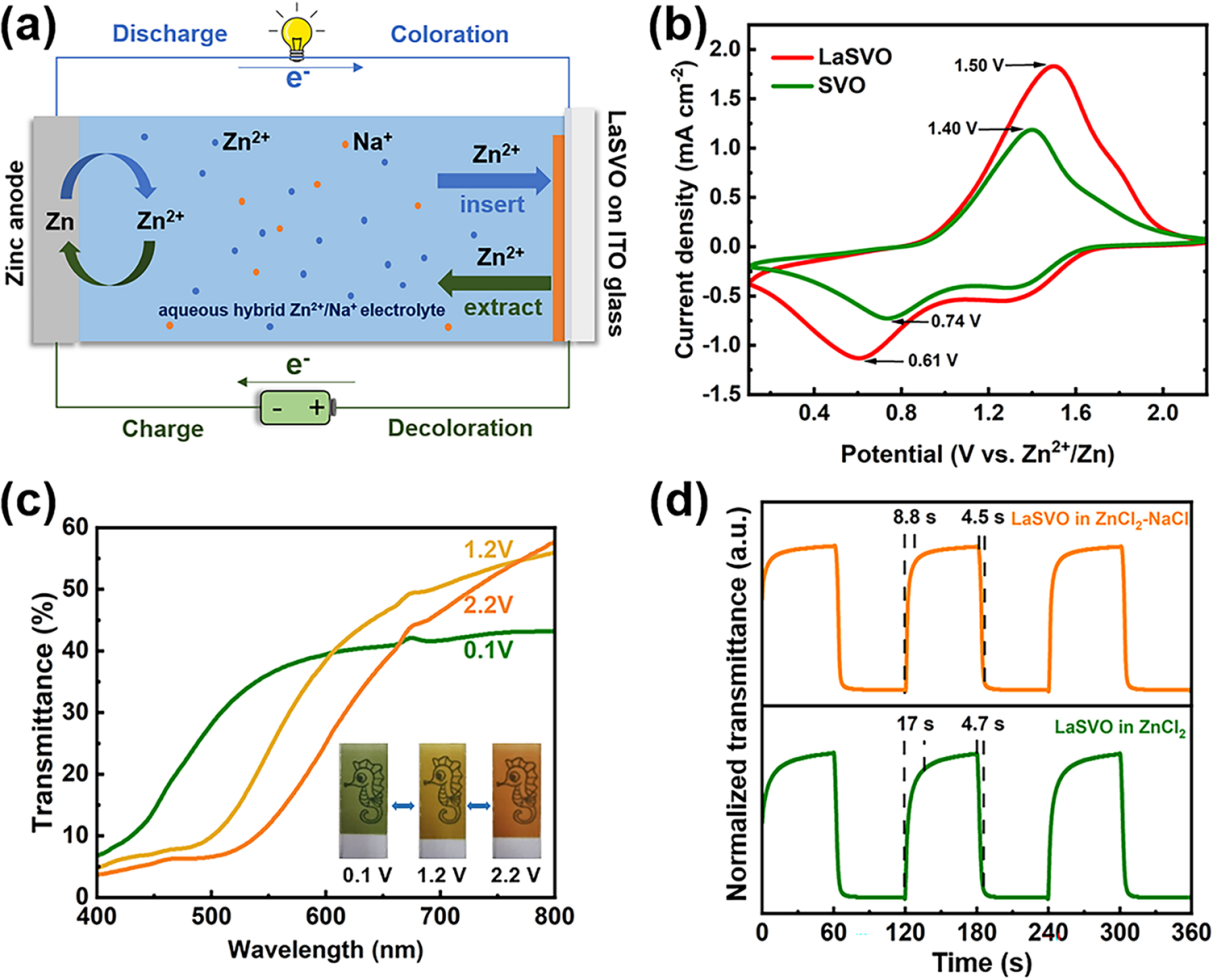
**a** Schematic diagram of Zn-LaSVO configuration. **b** Cyclic voltammograms of LaSVO and SVO in hybrid Zn^2+^/Na^+^ electrolyte, respectively, with a potential scan rate of 50 mV s^−1^. **c** Optical transmittance spectra of the LaSVO electrodes at different applied voltages in the hybrid Zn^2+^/Na^+^ electrolyte, inset: corresponding digital photos of the LaSVO electrodes. **d** The dynamic test of the LaSVO and SVO electrodes at 531 nm in the 0.1–2.2 V window

The working mechanism of the Zn-LaSVO two-electrode configuration is shown in Fig. 4a. Such a two-electrode configuration enables efficient energy retrieval while boosting the electrochromic performance of LaSVO through utilizing proper electrolyte systems [35, 36]. As depicted in Fig. 4b, the electrochemical activity of LaSVO in the aqueous hybrid Zn^2+^/Na^+^ electrolyte is significantly superior to that of SVO. A similar trend is also observed in the pure aqueous ZnCl_2_ electrolyte (Fig. S3). These results affirm that bimetallically doped vanadate (i.e., LaSVO) with wider interlayer spacing accelerates the transportation of cations.

To further evaluate the diffusion rate of zinc ions in LaSVO and SVO, we examined the current density evolution of LaSVO and SVO electrodes during CV testing at different scan rates (Fig. S4a, b). The ion diffusion coefficient was calculated and revealed according to the following Randles–Sevcik equation (Eq. 1):

1 $$ i_{p} = 2.687 \times 10^{5} \times n^{3/2} \times D^{1/2} \times C \times S \times v^{1/2} $$

where $i_{p}$ is the peak current (A), $n$ is the number of electrons participating in the reaction and is assumed to be 2, $D$ is the apparent ion diffusion coefficient (cm^2^ s^−1^), $C$ is the concentration of the active ion (Zn^2+^) in the electrolyte (mol cm^−3^), $S$ is the effective area of the LaSVO electrode (cm^2^), and $v$ is the potential scan rate (V s^−1^). To accurately figure out the Zn^2+^ ion diffusion coefficient in LaSVO and SVO electrodes, linear plots between peak currents from CV curves and the square root of the scan rates are illustrated in Fig. S4c. Accordingly, the diffusion coefficients of Zn^2+^ for intercalation and extraction of the LaSVO electrode are all calculated to be 1.98 × 10^−9^ cm^2^ s^−1^; whereas for the SVO electrode are 8.09 × 10^−10^ cm^2^ s^−1^ for intercalation and 1.26 × 10^−9^ cm^2^ s^−1^ for extraction (Fig. S4c). The higher diffusion coefficients of the LaSVO electrode are consistent with the wider interlayer spacing of LaSVO, indicating its excellent electrochromic performance.

Figure 4c shows the transmission spectra of the LaSVO electrode at different applied voltages in the aqueous hybrid Zn^2+^/Na^+^ electrolyte. The LaSVO electrode is orange, yellow, and green when being applied an external voltage of 2.2, 1.2 and 0.1 V, respectively. Moreover, the electrode is semitransparent at any applied voltages (the underlying pattern is visible, inset in Fig. 4c), indicating its promising applications in transparent optoelectronics. Furthermore, the switching times of the LaSVO tested in the aqueous hybrid Zn^2+^/Na^+^ electrolyte (4.5 s for coloration, 8.8 s for bleaching) are of significant advancement in comparison with that tested in aqueous pure ZnCl_2_ electrolyte (4.7 s for coloration, 17 s for bleaching). Remarkably, such switching times of LaSVO in the hybrid electrolyte are the fastest ones compared with state-of-the-art Zn-vanadate electrochromic displays [17, 24, 27]. Additionally, the coloration efficiency (CE) of the LaSVO electrode in the hybrid electrolyte, calculated as 70.74 cm^2^ C^−1^ (Fig. S5a), is higher than that tested in the pure ZnCl_2_ electrolyte (61.85 cm^2^ C^−1^, Fig. S5b). The value of 70.74 cm^2^ C^−1^ is also higher than other representative reports [17, 24, 37, 38], which further confirms the Zn//ZnCl_2_–NaCl//LaSVO electrochromic display platform a promising paradigm for energy-efficient transparent electrochromic displays. X-ray photoelectron spectroscopy (XPS) results reveal the color switching of LaSVO is originated from the oxidation and reduction of V (Fig. S6, Table S1), in a similar fashion to other vanadates [17, 24, 27].

Along with rapid switching times and high coloration efficiency realized by employing the aqueous hybrid Zn^2+^/Na^+^ electrolyte, the LaSVO electrode in the hybrid electrolyte demonstrates enhanced cycling stability in comparison with previous reports [17, 37–40]. The LaSVO electrode maintains 66.26% of initial capacity after 1000 CV cycles in the hybrid electrolyte, while only 21.49% of capacity is reserved in the pure ZnCl_2_ electrolyte after 1000 cycles (Fig. S7). Such a compelling cycling performance of LaSVO in the hybrid electrolyte is superior to the previously reported Zn-vanadate electrochromic displays [17, 24, 27]. Furthermore, we verified the excellent cycling stability of LaSVO electrodes in the hybrid electrolyte by investigating optical contrast retention. The results show that the LaSVO electrode maintains ~ 54% optical contrast after 1000 CV cycles in the hybrid electrolyte (Fig. S8a), while the LaSVO in the ZnCl_2_ electrolyte was almost fully dissolved (Fig. S8b). Likewise, the LaSVO electrode exhibits better cycle-to-cycle stability in the hybrid electrolyte than in the pure ZnCl_2_ electrolyte. As shown in Fig. S8c, the LaSVO electrode maintains 68.1% optical contrast after 1000 switching cycles (much better than the results investigated in the ZnCl_2_ electrolyte, Fig. S8d).

### LaSVO-Zn-LaSVO Transparent Electrochromic Displays

**Fig. 5:**
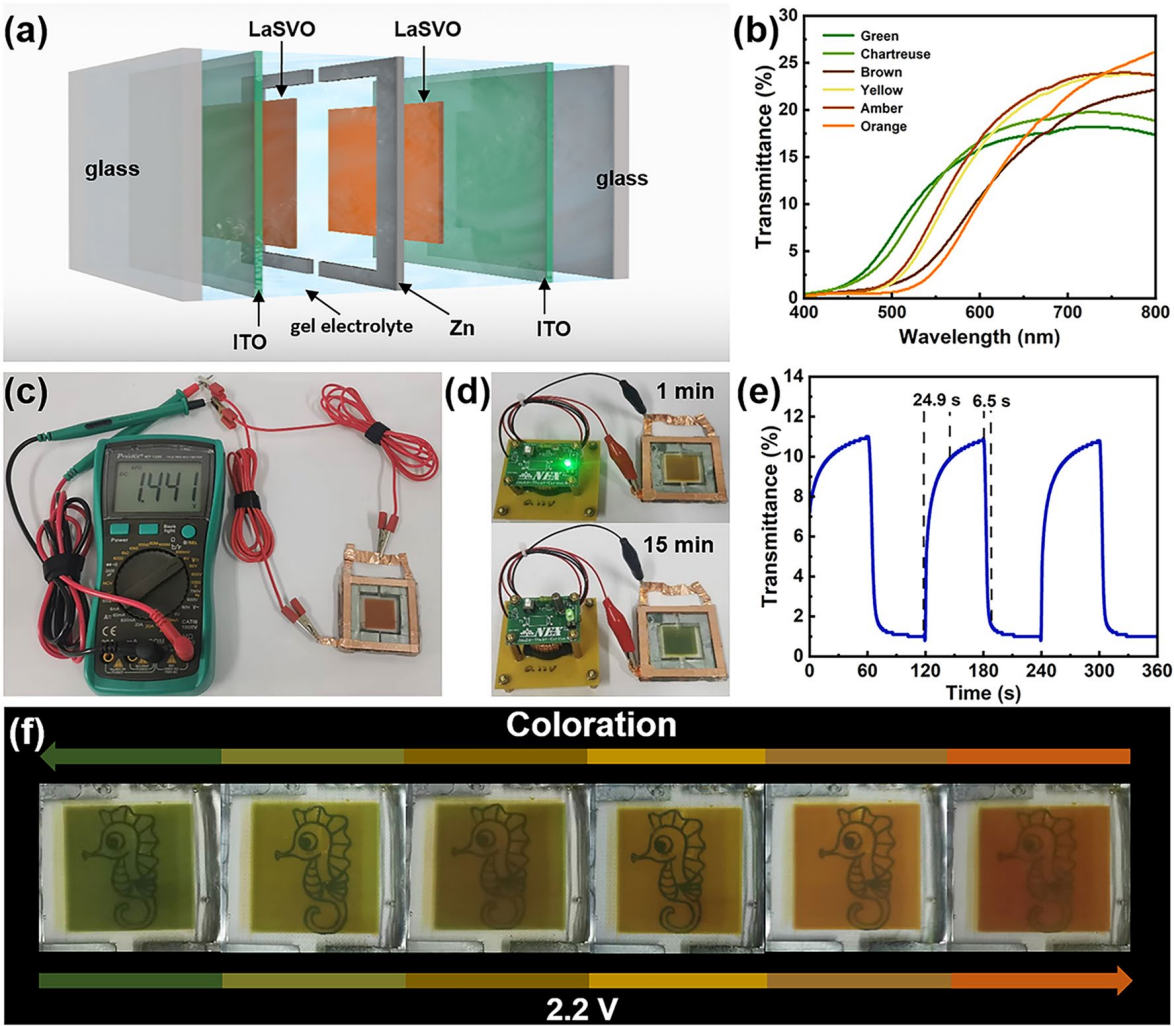
**a** Schematic diagram of the LaSVO-Zn-LaSVO electrochromic display configuration. **b** Optical transmittance spectra of the LaSVO-Zn-LaSVO electrochromic display at different applied voltages. **c** Digital photograph of the orange-colored LaSVO-Zn-LaSVO electrochromic display, showing an OCP of 1.44 V. **d** Digital photographs of a 0.2 V regulated LED powered by the LaSVO-Zn-LaSVO electrochromic display at 1 and 15 min. **e** The dynamic test of the LaSVO-Zn-LaSVO electrochromic display at 531 nm in the 0.1–2.2 V electrochemical window. **f** Digital photographs of the LaSVO-Zn-LaSVO transparent electrochromic display with various color variations, which clearly show the cartoon pattern underneath the display

To demonstrate the applicability of the Zn//ZnCl_2_-NaCl//LaSVO electrochromic display platform, a 5 cm × 5 cm transparent multicolor electrochromic display is assembled using a PVP-based gel electrolyte (see Sect. 2), as depicted in Fig. 5a. Since the LaSVO electrodes having intermediate colors between orange and green, the build of LaSVO-Zn-LaSVO platform enables a richer color palette via superimposing the intermediate colors of two LaSVO segments [17, 24]. As shown in Fig. 5b, the LaSVO-Zn-LaSVO display exhibits various colors due to the color overlay of the intermediate colors of LaSVO. In addition, the redox potential difference between the Zn anode and orange colored-LaSVO cathode results in an open circuit potential (OCP) of 1.44 V (Fig. 5c) [41], which enables the spontaneous color switching from orange to green via lighting a 0.2 V regulated LED for 15 min (Fig. 5d). The dynamic transmittance characteristics of the LaSVO-Zn-LaSVO electrochromic display were evaluated in the 0.1–2.2 V window and are shown in Fig. 5d, where the switching times are calculated to be 24.9 s for bleaching and 6.5 s for coloration. While the switching times of the prototype display are inferior to that of a single LaSVO electrode (due to the large areal effect and the decay of the ionic conductivity resulting from the gel electrolyte), the switching times of the current display are superior to the previous reports [17, 24]. Furthermore, the LaSVO-Zn-LaSVO electrochromic display has excellent semitransparency, allowing the underlying cartoon to be visible to the naked eye (Fig. 5f).

## Conclusions

In summary, this study demonstrates the successful preparation of bimetallically doped vanadate (i.e., LaSVO) through a simple liquid–solid stirring method for the first time. This innovative approach significantly broadens the interlayer spacing between V_3_O_8_ layers without changing the phase structure of SVO, resulting in an improved ionic diffusion coefficient. By introducing Na^+^ into the Zn^2+^-electrolyte, the dissolution equilibrium of Na^+^ from LaSVO is effectively modified, leading to substantial inhibition of LaSVO dissolution in the aqueous electrolyte. In addition, the inclusion of Na^+^ in the electrolyte proves to be highly effective in preventing the formation of Zn dendrites, which enables high reversibility of the Zn anode. These results, including both cathode materials and electrolyte composition design, successfully solve the challenges concerned in vanadate cathode and Zn anode. As a result, the advances presented in this article are expected to accelerate the development of electrochromic displays.

### Supplementary Information

Below is the link to the electronic supplementary material.

Supplementary file1 (PDF 1161 kb)

## References

[1] Gu C, Jia AB, Zhang YM, Zhang SXA (2022). Emerging electrochromic materials and devices for future displays. Chem. Rev..

[2] Wu W, Fang H, Ma H, Wu L, Zhang W (2021). Boosting transport kinetics of ions and electrons simultaneously by Ti_3_C_2_T_x_ (MXene) addition for enhanced electrochromic performance. Nano-Micro Lett..

[3] Fu H, Zhang L, Dong Y, Zhang C, Li W (2023). Recent advances in electrochromic materials and devices for camouflage applications. Mater. Chem. Front..

[4] Chen K, He J, Zhang D, You L, Li X (2021). Bioinspired dynamic camouflage from colloidal nanocrystals embedded electrochromics. Nano Lett..

[5] Fu H, Yan S, Yang T, Yin M, Zhang L (2022). New dual conjugated polymer electrochromic device with remarkable yellow-to-green switch for adaptive camouflage. Chem. Eng. J..

[6] Wang B, Huang Y, Han Y, Zhang W, Zhou C (2022). A facile strategy to construct Au@V_x_O_2x+1_ nanoflowers as a multicolor electrochromic material for adaptive camouflage. Nano Lett..

[7] Hartel MC, Lee D, Weiss PS, Wang J, Kim J (2022). Resettable sweat-powered wearable electrochromic biosensor. Biosens. Bioelectron..

[8] Yun TG, Park M, Kim D-H, Kim D, Cheong JY (2019). All-transparent stretchable electrochromic supercapacitor wearable patch device. ACS Nano.

[9] Wang C, Jiang X, Cui P, Sheng M, Gong X (2021). Multicolor and multistage response electrochromic color-memory wearable smart textile and flexible display. ACS Appl. Mater. Interfaces.

[10] Singh SB, Tran DT, Jeong KU, Kim NH, Lee JH (2022). A flexible and transparent zinc-nanofiber network electrode for wearable electrochromic, rechargeable Zn-ion battery. Small.

[11] Jiao X, Wang J, Yuan Z, Zhang C (2023). Smart current collector for high-energy-density and high-contrast electrochromic supercapacitors toward intelligent and wearable power application. Energy Storage Mater..

[12] Bessinger D, Muggli K, Beetz M, Auras F, Bein T (2021). Fast-switching Vis–IR electrochromic covalent organic frameworks. J. Amer. Chem. Soc..

[13] Lahav M, van der Boom ME (2018). Polypyridyl metallo-organic assemblies for electrochromic applications. Adv. Mater..

[14] Yang M, Zhao R, Zhang S, Wang L, Zhang Z (2023). Facile synthesis of V_2_O_5_ films and devices exhibiting multicolor electrochromic properties. Mater. Sci. Eng. B.

[15] Li W, Han C, Gu Q, Chou S-L, Wang J-Z (2020). Electron delocalization and dissolution-restraint in vanadium oxide superlattices to boost electrochemical performance of aqueous zinc-ion batteries. Adv. Energy Mater..

[16] Zhao Q, Pan Z, Liu B, Bao C, Liu X (2023). Electrochromic-induced rechargeable aqueous batteries: an integrated multifunctional system for cross-domain applications. Nano-Micro Lett..

[17] Zhang W, Li H, Yu WW, Elezzabi AY (2020). Transparent inorganic multicolour displays enabled by zinc-based electrochromic devices. Light Sci. Appl..

[18] Zhang D, Cao J, Yue Y, Pakornchote T, Bovornratanaraks T (2021). Two birds with one stone: Boosting zinc-ion insertion/extraction kinetics and suppressing vanadium dissolution of V_2_O_5_ via La^3+^ incorporation enable advanced zinc-ion batteries. ACS Appl. Mater. Interfaces.

[19] Deng C, Zhang K, Liu L, He Z, Huang J (2022). High-performance all-solid-state electrochromic asymmetric Zn-ion supercapacitors for visualization of energy storage devices. J. Mater. Chem. A.

[20] Wu C, Shi H, Zhao L, Chen X, Zhang X (2023). High-performance aqueous Zn^2+^/Al^3+^ electrochromic batteries based on niobium tungsten oxides. Adv. Funct. Mater..

[21] Ren R, Liu S, Gao Y, Lei P, Wang J (2023). Tunable interaction between Zn^2+^ and superstructured Nb_18_W_16_O_93_ bimetallic oxide for multistep tinted electrochromic device. ACS Energy Lett..

[22] Wu Z, Lian Z, Yan S, Li J, Xu J (2022). Extraordinarily stable aqueous electrochromic battery based on Li_4_Ti_5_O_12_ and hybrid Al^3+^/Zn^2+^ electrolyte. ACS Nano.

[23] Liu L, Zhen M, Wang L, Li B, Deng C (2023). Full-temperature all-solid-state dendrite-free Zn-ion electrochromic energy storage devices for intelligent applications. Chem. Eng. J..

[24] Wang B, Zhao F, Zhang W, Li C, Hu K (2023). Inhibiting vanadium dissolution of potassium vanadate for stable transparent electrochromic displays. Small Sci..

[25] Zhang W, Li H, Elezzabi AY (2022). Electrochromic displays having two-dimensional CIE color space tunability. Adv. Funct. Mater..

[26] Liang Y, Cao S, Wei Q, Zeng R, Zhao J (2021). Reversible Zn^2+^ insertion in tungsten ion-activated titanium dioxide nanocrystals for electrochromic windows. Nano-Micro Lett..

[27] Zhang W, Li H, Al-Hussein M, Elezzabi AY (2020). Electrochromic battery displays with energy retrieval functions using solution-processable colloidal vanadium oxide nanoparticles. Adv. Opt. Mater..

[28] Gu X, Wang J, Zhao X, Jin X, Jiang Y (2023). Engineered nitrogen doping on VO_2_(B) enables fast and reversible zinc-ion storage capability for aqueous zinc-ion batteries. J. Energy Chem..

[29] Li H, Li J, Hou C, Ho D, Zhang Q (2017). Solution-processed porous tungsten molybdenum oxide electrodes for energy storage smart windows. Adv. Mater. Technol..

[30] Wan F, Zhang L, Dai X, Wang X, Niu Z (2018). Aqueous rechargeable zinc/sodium vanadate batteries with enhanced performance from simultaneous insertion of dual carriers. Nat. Commun..

[31] Huang S, Zhu J, Tian J, Niu Z (2019). Recent progress in the electrolytes of aqueous zinc-ion batteries. Chem. Eur. J..

[32] Gao Y, Liu Z, Guo S, Cao X, Fang G (2022). Fundamental understanding and effect of anionic chemistry in zinc batteries. Energy Environ. Mater..

[33] Wang C, Pei Z, Meng Q, Zhang C, Sui X (2021). Toward flexible zinc-ion hybrid capacitors with superhigh energy density and ultralong cycling life: the pivotal role of ZnCl_2_ salt-based electrolytes. Angew. Chem. Int. Ed..

[34] Su L, Liu L, Liu B, Meng J, Yan X (2020). Revealing the impact of oxygen dissolved in electrolytes on aqueous zinc-ion batteries. iScience.

[35] Zhao F, Wang B, Zhang W, Cao S, Liu L (2023). Counterbalancing the interplay between electrochromism and energy storage for efficient electrochromic devices. Mater. Today.

[36] Zhang W, Li H, Yu WW, Elezzabi AY (2021). Emerging Zn anode-based electrochromic devices. Small Sci..

[37] Liu Y, Jia C, Wan Z, Weng X, Xie J (2015). Electrochemical and electrochromic properties of novel nanoporous NiO/V_2_O_5_ hybrid film. Sol. Energy Mater. Sol. Cells.

[38] He W, Liu Y, Wan Z, Jia C (2016). Electrodeposition of V_2_O_5_ on TiO_2_ nanorod arrays and their electrochromic properties. RSC Adv..

[39] Mjejri I, Rougier A, Gaudon M (2017). Low-cost and facile synthesis of the vanadium oxides V_2_O_3_, VO_2_, and V_2_O_5_ and their magnetic, thermochromic and electrochromic properties. Inorg. Chem..

[40] Tang K, Zhang Y, Shi Y, Cui J, Shu X (2018). Preparation of V_2_O_5_ dot-decorated WO_3_ nanorod arrays for high performance multi-color electrochromic devices. J. Mater. Chem. C.

[41] Zhang W, Li H, Elezzabi AY (2023). A dual-mode electrochromic platform integrating zinc anode-based and rocking-chair electrochromic devices. Adv. Funct. Mater..

