# Supplementary material for: Fast and Stable Zinc Anode-Based Electrochromic Displays Enabled by Bimetallically Doped Vanadate and Aqueous Zn2+/Na+ Hybrid Electrolytes
Source: Nanomicro Lett. 2023 Oct 17;15:229. doi: 10.1007/s40820-023-01209-z (PMC10581958; doi:10.1007/s40820-023-01209-z)
Supplement: Supplementary file 1 — Supplementary file1 (PDF 1161 kb) [file 40820_2023_1209_MOESM1_ESM.pdf]

Supporting Information for

Fast and Stable Zinc Anode-Based Electrochromic Displays Enabled by Bimetallically Doped Vanadate and Aqueous Zn^{2+}/Na^{+} Hybrid Electrolytes

Zhaoyang Song[1, 2, #], Bin Wang[2, #], Wu Zhang[4], Qianqian Zhu[1, *], Abdulhakem Y. Elezzabi[4], Linhua Liu[2], William W. Yu[3], and Haizeng Li[2, *]

[1]College of Chemistry and Chemical Engineering, Qingdao University, Qingdao 266071, People's Republic of China
[2]Optics & Thermal Radiation Research Center, Institute of Frontier and Interdisciplinary Science, Shandong University, Qingdao 266237, People's Republic of China

[3]School of Chemistry & Chemical Engineering, Shandong University, Jinan 250100, People's Republic of China

[4]Ultrafast Optics and Nanophotonics Laboratory, Department of Electrical and Computer Engineering, University of Alberta, Edmonton, Alberta T6G 2V4, Canada

[#] Zhaoyang Song and Bin Wang contributed equally to this work.
*Corresponding authors. E-mail: zhuqianqian@qdu.edu.cn (Qianqian Zhu), haizeng@sdu.edu.cn (Haizeng Li)

Supplementary Figures and Tables

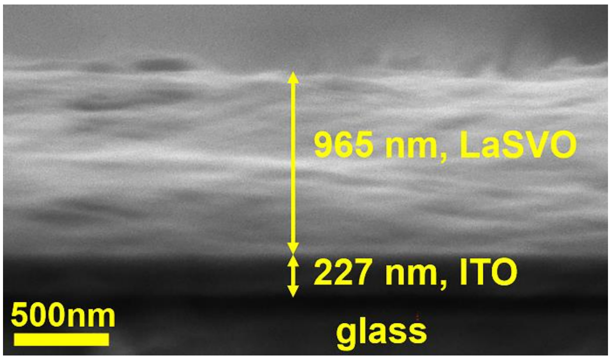

Fig. S1 Cross-sectional SEM image of the LaSVO electrode

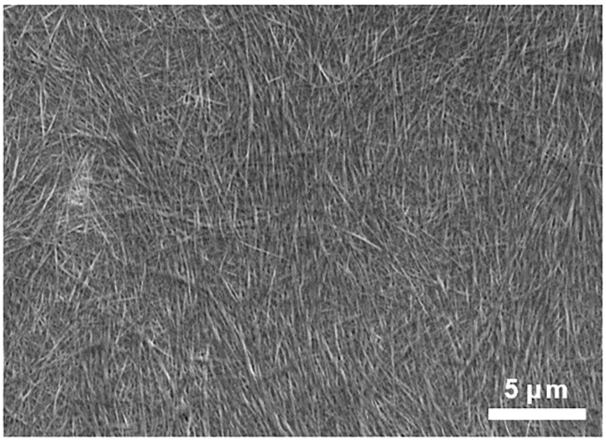

Fig. S2 SEM image of SVO nanofibers

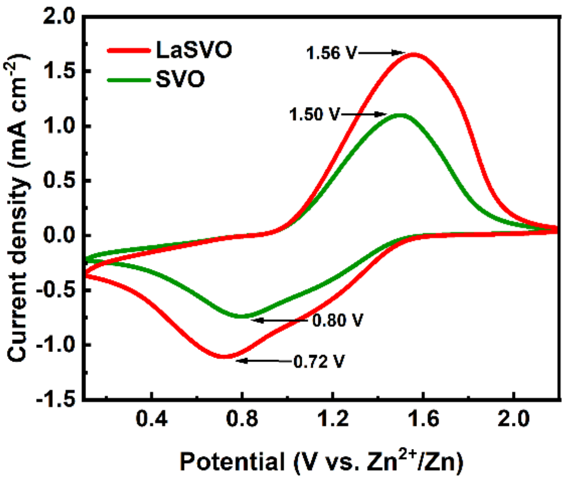

Fig. S3 Cyclic voltammograms of LaSVO and SVO electrodes in 1M $ZnCl_2$ electrolyte, with a potential scan rate of 50 mV s^{-1}

Figure S3 shows that the electrochemical activity of the LaSVO electrode is greater than that of the SVO electrode in 1M $ZnCl_2$ electrolyte.

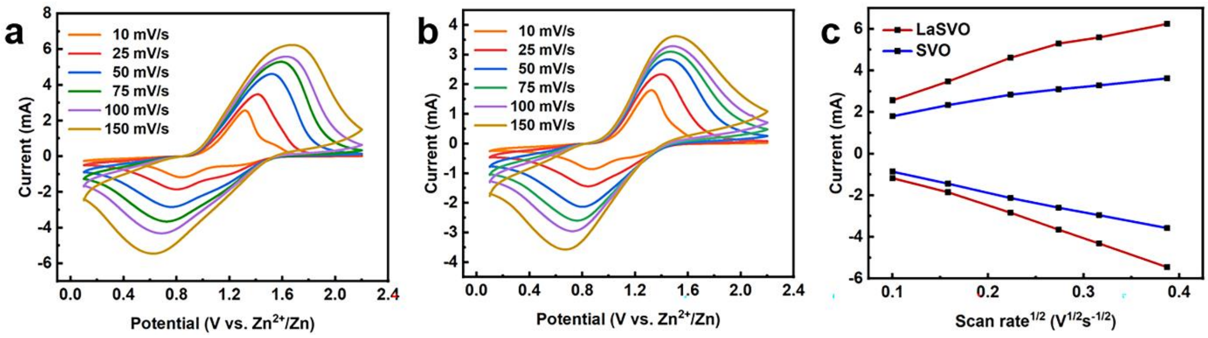

Fig. S4 a Cyclic voltammetry test of LaSVO electrode at different scan rates in 1 M $ZnCl_2$ electrolyte. **b** Cyclic voltammetry test of SVO electrode at different scan rates in 1 M $ZnCl_2$ electrolyte. **c** The relationship between the peak current and the square root of scan rate for LaSVO and SVO at different scan rates in 1 M $ZnCl_2$ electrolyte

As shown in Fig. S4c, there is an approximately linear relationship between the peak current and the square root of scan rate for LaSVO and SVO at different scan rates in 1 M $ZnCl_2$ electrolyte. The slope of linear plots is related to the apparent ion diffusion coefficient (D), according to the Randles-Sevcik equation (Eq. (1) shown in main text). It clearly reveals that the Zn^{2+} diffusion coefficient of LaSVO (1.98×10^{-9} cm^2 s^{-1} for intercalation and 1.98×10^{-9} cm^2 s^{-1} for extraction) is high than that of SVO (8.09×10^{-10} cm^2 s^{-1} for intercalation and 1.26×10^{-9} cm^2 s^{-1} for extraction). These results are originated from the enlarged interlayer spacing of LaSVO.

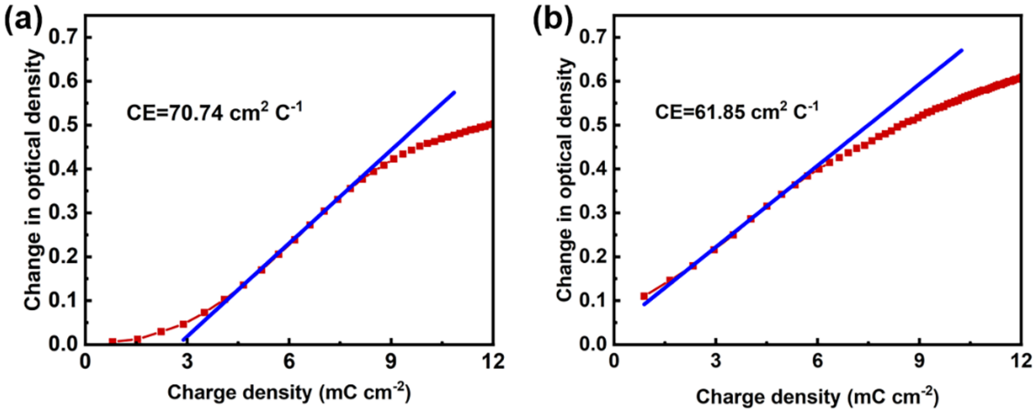

Fig. S5 Coloration efficiency of LaSVO in (a) the hybrid electrolyte and (b) the pure $ZnCl_2$ electrolyte

The coloration efficiency (CE) is calculated according to the following equation (Eq. (S1)):

$$CE = \frac{\Delta OD}{\Delta Q} = \frac{\log(T_b/T_c)}{\Delta Q}, \qquad (S1)$$

where T_b and T_c are the transmittances in bleached and colored states at specific wavelengths, respectively, and ΔQ is the injected charge. The CE value of the LaSVO electrode in 1 M $ZnCl_2$ solution is calculated to be 61.85 cm^2 C^{-1} (Fig. S5b), which is smaller than the LaSVO electrode in the hybrid electrolyte (70.74 cm^2 C^{-1}, Fig. S5a).

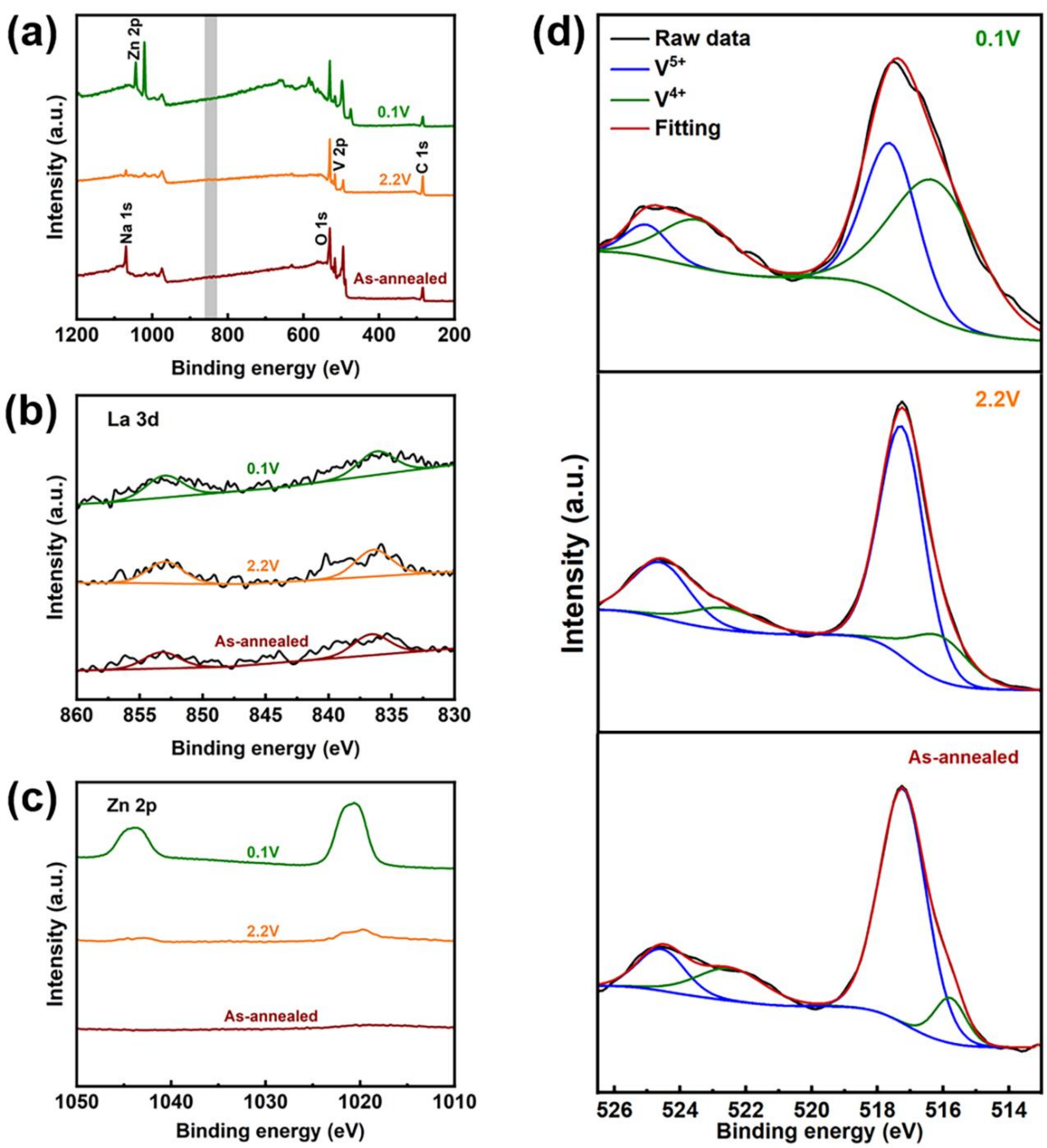

Fig. S6 The ex-situ XPS survey spectra of LaSVO electrode at different states: **a** full survey. **b** La 3d. **c** Zn 2p. **d** V 2p

Figure S6a shows the ex-situ full measurement spectra of the LaSVO electrode under different applied voltages. Compared to the as-annealed LaSVO electrodes, the weak Na 1s peaks observed in the oxidation and reduction states can be attributed to the strong electrostatic interaction and high charge density of the Zn^{2+} cation. The large charge density of Zn^{2+} cations could facilitate the formation of bonds between Zn^{2+} and oxygen atoms in vanadates, thus enabling the extrusion of inserted Na^{+} from LaSVO [S1]. Figure S6b shows the high-resolution La 3d nucleolar XPS spectra, which confirm the presence of La element. The two peaks are located near 836 and 853 eV, corresponding to La $3d_{5/2}$ and La $3d_{3/2}$ [S2, S3], respectively. Fig. S6c shows that the as-annealed LaSVO electrode does not contain Zn element, while the reduced LaSVO electrode (i.e., LaSVO electrode after 0.1V discharge) has obvious Zn element peaks.

This indicates the intercalation of Zn^{2+} during the discharging process. Notably, only ~4.4% of the inserted Zn^{2+} resides in the 2.2 V-charged LaSVO electrode (calculation process outlined in Note S1, Supporting Information), indicating that the LaSVO electrode has excellent reversibility. Fig. S6d shows the high-resolution V 2p core-level XPS spectra of LaSVO films at different states (discharge/charge/as-annealed). The inserting/extracting of zinc ions results in the valence state change of vanadium. The two most intense pairs of peaks, located at 517.2 and 524.6 eV, are attributed to V^{5+}. Another pair of peaks centered at 516.2 and 523.2 eV corresponds to V^{4+} [S4].

Note S1: Calculation Process of Zn^{2+} Content in a LaSVO Electrode

Firstly, the XPS spectrum is subjected to a peak splitting operation by the software Avantage to obtain the corresponding areas of the Zn 2p peaks. Secondly, the normalized area of the Zn 2p peaks is calculated according to the equation:

$$\text{Normalized Area [Area (N)]} = \text{Peak Area} / (\text{SF} * \text{TXFN} * \text{ECF}) \qquad \text{(S2)}$$

TXFN = transmission function

SF = sensitivity factor

ECF = energy compensation factor

According to Eq. (S2), the normalized area of Zn 2p in the reduced and oxidized LaSVO electrode is calculated as 4320.75 and 191.31, respectively. Therefore, only ~4.4% of the inserted Zn^{2+} resides in the LaSVO electrode by applying a 2.2 V external voltage.

Table S1 The atomic ratio of V^{4+} and V^{5+} in reduced (0.1 V), oxidized (2.2 V), and as-annealed LaSVO films

**Sample**	**The atomic percentage of V in different valence states**		**V^{4+}/V^{5+}**
	**V^{4+}**	**V^{5+}**	
0.1 V	0.621	0.379	1.64
2.2 V	0.232	0.768	0.30
As-annealed	0.221	0.779	0.28

Table S1 shows that the atomic number ratio of V^{4+}/V^{5+} of the 0.1V-discharged LaSVO electrode is 1.64, and the atomic number ratio of V^{4+}/V^{5+} of the 2.2V-charged LaSVO electrode is 0.30. These results indicate that the color switching of Zn-LaSVO electrochromic devices is attributed to the ratio change of V^{4+}/V^{5+}.

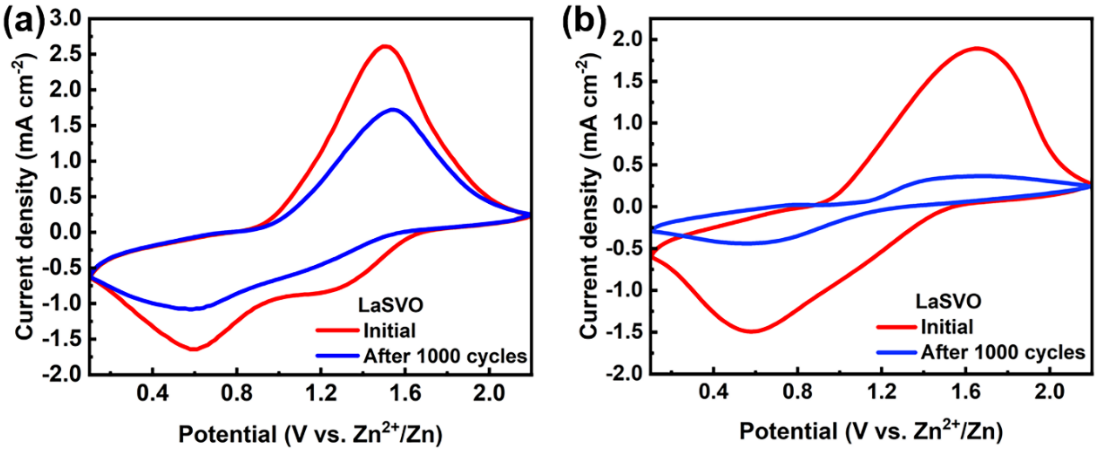

Fig. S7 Cyclic voltammetry stability of the LaSVO electrode tested in **(a)** the aqueous Zn^{2+}/Na^+ hybrid electrolyte and **(b)** the pure $ZnCl_2$ electrolyte at a scan rate of 100 mV s^{-1}.

The LaSVO electrode maintains 66.26% of initial capacity after 1000 CV cycles in the hybrid electrolyte (Fig. S7a), while only 21.49% of capacity is reserved in the pure $ZnCl_2$ electrolyte after 1000 cycles (Fig. S7b).

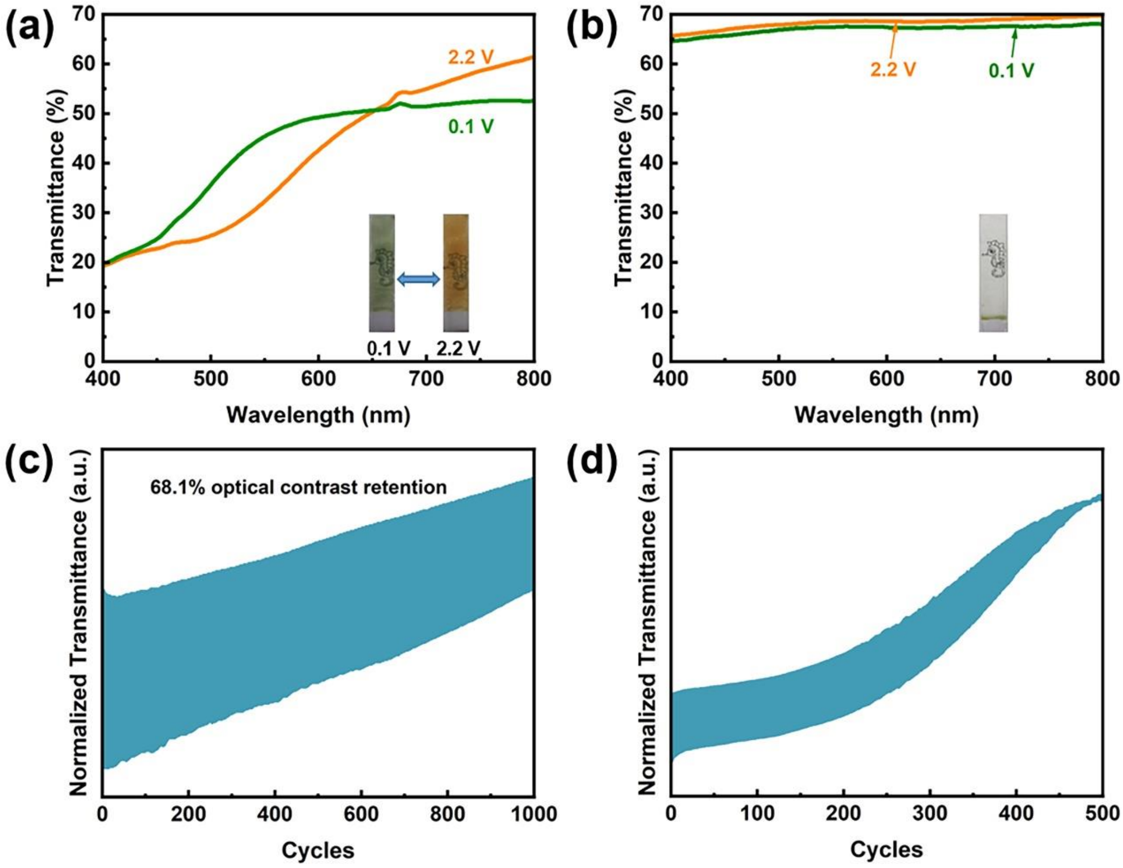

Fig. S8 a, b Transmission spectra and digital photographs of LaSVO electrodes after 1000 CV cycles in the (a) hybrid electrolyte and (b) pure $ZnCl_2$ electrolyte. **c, d** Cycling durability profiles of LaSVO electrodes in the (c) hybrid electrolyte and (d) pure $ZnCl_2$ electrolyte under 2.2 V and 0.1 V switching for 15 s intervals

The LaSVO electrode retained ~54% optical contrast after 1000 CV cycles in the hybrid electrolyte (Fig. S8a) and was almost fully dissolved in the $ZnCl_2$ electrolyte (Fig. S8b). The LaSVO electrode in the hybrid electrolyte maintained 68.1% optical contrast after 1000 switches (Fig. S8c), which was much better than that in the $ZnCl_2$ electrolyte (Fig. S8d).

Supplementary References

[S1] X. Guo, G. Fang, W. Zhang, J. Zhou, L. Shan, L. Wang, C. Wang, T. Lin, Y. Tang, S. Liang, Mechanistic insights of Zn^{2+} storage in sodium vanadates. Adv. Energy Mater. **8**(27), 1801819 (2018). https://doi.org/10.1002/aenm.201801819.

[S2] J. Yu, C. Li, B. Li, X. Zhu, R. Zhang, L. Ji, D. Tang, A. M. Asiri, X. Sun, Q. Li, S. Liu, Y. Luo. A perovskite $La_2Ti_2O_7$ nanosheet as an efficient electrocatalyst for artificial N_2 fixation to NH_3 in acidic media. Chem. Commun. **55**(45), 6401-6404 (2019). https://doi.org/10.1039/C9CC02310K

[S3] J. He, X. Liu, H. Zhang, Z. Yang, X. Shi, Q. Liu, X. Lu. Enhancing Zn-ion storage capability of hydrated vanadium pentoxide by the strategic introduction of La^{3+}. ChemSusChem. **13**(6), 1568-1574 (2020). https://doi.org/10.1002/cssc.201902659

[S4] G. Silversmit, D. Depla, H. Poelman, G. B. Marin, R. De Gryse. Determination of the V2p XPS binding energies for different vanadium oxidation states (V^{5+} to V^{0+}). J. Electron. Spectrosc. Relat. Phenom. **135**(2), 167-175 (2004). https://doi.org/10.1016/j.elspec.2004.03.004
